# Pulmonary tumor embolism in renal cell carcinoma detected by hybrid CT and F18-PSMA PET

**DOI:** 10.1016/j.radcr.2023.08.090

**Published:** 2023-09-19

**Authors:** Jared E. Tan, Sai Vishnu, Dalveer Singh

**Affiliations:** a The Melbourne Medical School, University of Melbourne, Melbourne, Victoria, Australia; b Qscan Radiology Clinics, Woolloongabba, Queensland, Australia; c School of Medicine, University of Queensland, Brisbane, Queensland, Australia

**Keywords:** Pulmonary tumor embolism, Renal cell carcinoma, PSMA PET/CT

## Abstract

We present the case of a 75-year-old female in which a pulmonary tumor embolism was detected incidentally on a prostate-specific membrane antigen positron emission tomography-computed tomography restaging scan. This occurred on the background of renal cell carcinoma in remission with pazopanib systemic therapy and a right nephrectomy 4 years prior. An avidity to prostate-specific membrane antigen in the superior lingula of the left upper lobe of the lung coupled with contrast-enhanced computed tomography findings found the lesion to be a tumor thrombus. This case serves to highlight the effectiveness of incorporating contrast-enhanced computed tomography with prostate-specific membrane antigen positron emission tomography and to consider the rare diagnosis of a pulmonary tumor embolism.

## Introduction

The development of novel second-generation radiotracers targeting prostate-specific membrane antigens (PSMA) has led to increased diagnostic and staging capacity in both prostatic and non-prostatic cancers. An emerging gold standard is 2-(3-{1-carboxy-5-[(6-18F-fluoro-pyridine -3-carbonyl)-amino]-pentyl}-ureido)-pentanedioic acid (18F-DCFPyL), and when used in combination with positron emission tomography-computed tomography (PET/CT) hybrid imaging, it bestows the clinician tremendous diagnostic ability [1]. But with increased diagnostic power comes the responsibility to detect rare, previously disregarded lesions. This case report describes the incidental detection of a pulmonary tumor embolism (PTE) in a dyspneic patient with renal cell carcinoma (RCC) upon routine PSMA-PET/CT staging. To our knowledge, this is the only case report describing a PTE in a patient with RCC detected by PSMA-PET/CT.

## Case report

**Fig. 1:**
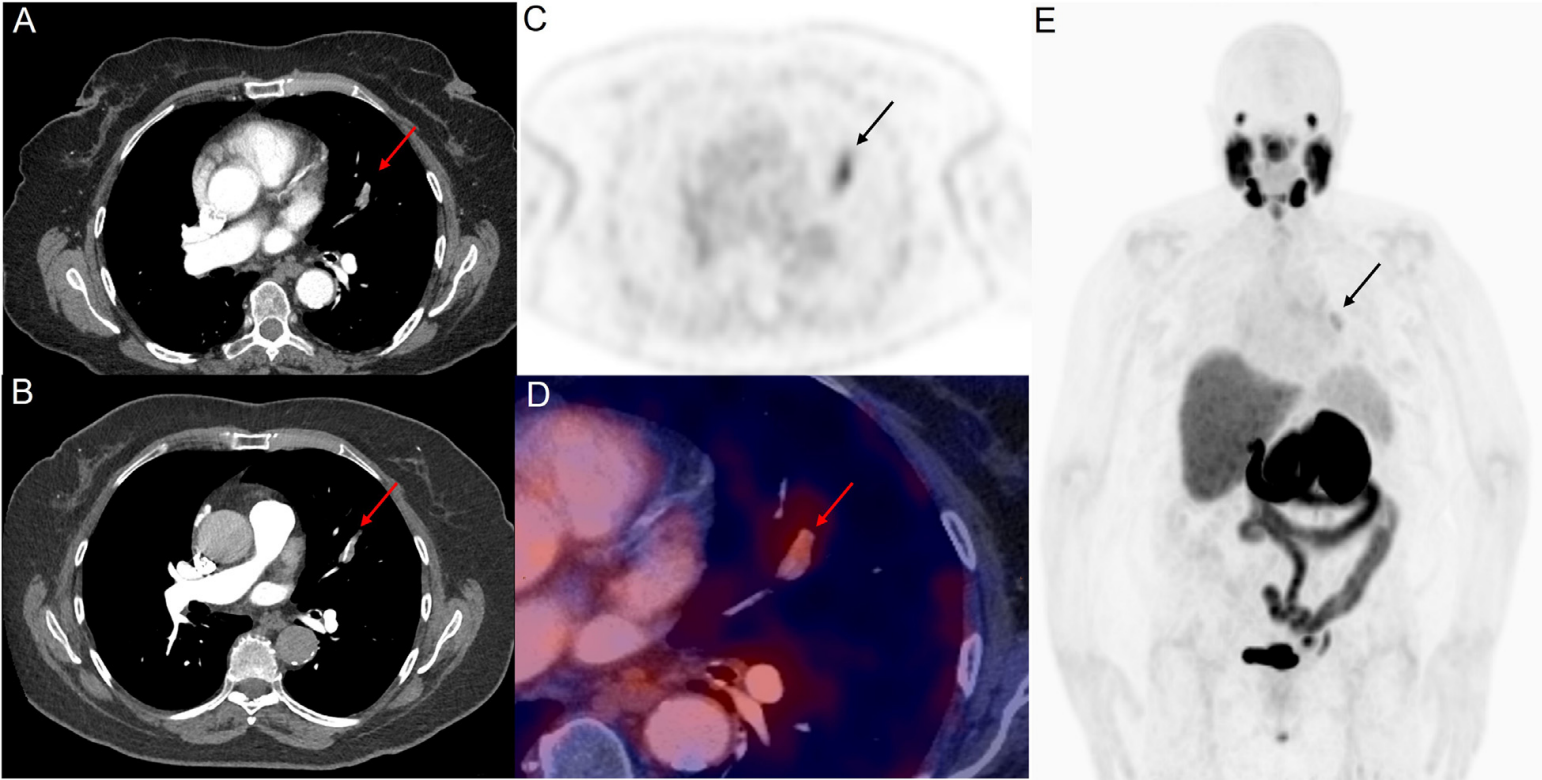
Hybrid contrast-enhanced CT (with iodinated contrast bolus timed to maximize pulmonary arterial visualization) and 18F-DCFPyL PSMA PET images. Contrast CT images (A, B) show an eccentric filling defect in the superior lingula segmental artery of the left lung upper lobe (red arrow). This is concordant with moderately intense PSMA radiotracer expression (black arrows on axial PET only images, C and on whole body maximum intensity projection image, E). Fused PET and contrast-enhanced CT axial image (D) also confirm the lesion (red arrow). No other site of PSMA expressing metastasis is evident on whole-body imaging (E).

A 75-year-old female presented with recent onset dyspnea and left-sided chest pain. Her history included a right nephrectomy for clear renal cell carcinoma which had renal vein involvement (pT3N0) 4 years earlier. Previous staging imaging had demonstrated thoracic mediastinal nodal metastases and she was subsequently treated with pazopanib systemic therapy (tyrosine kinase inhibitor). She had a complete radiological response based on serial contrast-enhanced CT and 18F-DCFPyL PET imaging and the patient had then been off treatment given remission. Given her new onset of symptoms, restaging combined hybrid contrast-enhanced CT (with iodinated contrast bolus timed to maximize pulmonary arterial visualization) and 18F-DCFPyL PSMA PET was performed. Hybrid imaging demonstrated focal abnormal PSMA tracer expression in the superior lingula segment of the left upper lobe of the lung. This was confirmed on high-resolution fused PET/CT images to be within an eccentric filling defect in the lingula segmental pulmonary artery (Fig. 1). Given the PSMA expression, this was interpreted as a new metastatic tumor thrombus. No other site of PSMA expressing metastatic disease was evident. Subsequent re-introduction of pazopanib tyrosine kinase inhibitor therapy led to subsequent regression of the lingula segment lesion on subsequent CT imaging. The patient's chest pain improved and dyspnea gradually improved and has had clear CT surveillance imaging 6 months post initial presentation.

## Discussion

Renal cell carcinoma (RCC) is the most common cancer of the kidney and accounts for roughly 2% of global cancer diagnoses [2]. A crucial element of cancer management is staging which ascertains disease extent, and presence of distant metastases, and establishes the viability of available treatment options.

Prostate-specific membrane antigen is a transmembrane glycoprotein that is classically overexpressed in cancerous prostate cells [3]. Despite the name, expression of PSMA is not limited to prostate cancer. RCC subtypes (predominantly clear cell) in addition to the endothelium of tumor-associated neovasculature have also been shown to overexpress PSMA [4]. Utilizing this knowledge, PSMA-targeting radiotracers, such as 18F-DCFPyL and Ga68-PSMA-HBED-CC, have been developed which yield a greater degree of sensitivity and specificity than conventional PET functional imaging agents like 18F-FDG and 18F-NaF [5]. Among the novel PSMA-targeting agents, F-18 PSMA agents show superior spatial resolution capacity over Ga-68 agents [5]. Used in conjunction with hybrid imaging, such as PET/CT, novel PSMA radiotracers afford monumental benefits in assessing tumor spread, treatment response, and recurrence [6].

In this case report, the patient displayed pulmonary PSMA-avidity on PET/CT. Naturally, with PSMA-avidity at the lungs on staging PET/CT, one may be inclined to presume pulmonary metastases, especially if nodular in appearance and numerous. However, given the exhaustive list of pathology that expresses PSMA, this presumption cannot be made necessarily. Inflammatory and infectious lung conditions can display PSMA-avidity and thereby mimic potential pulmonary metastases [7]. Furthermore, given that PSMA can be constitutively expressed in nonprostatic noncancerous tissues under physiological conditions, the utility of targeting PSMA can become somewhat equivocal. To mitigate confounding, diagnostic contrast-enhanced CT (CECT) can be used as a point of differentiation, especially when delineating physiological from pathological PSMA-avidity [8]. This highlights the importance of PET being coupled with CECT compared to conventional nondiagnostic CT alone with PET for attenuation correction. This is further punctuated in rarer conditions such as pulmonary tumor embolism (PTE) where CECT is the sole method of detection.

RCC is a malignancy associated with tumor thrombus formation where 4-10% of patients possess a degree of tumor extension into the renal vein or inferior vena cava [9]*.* Autopsy studies revealed its presence in 3%-26% among patients with solid tumors [10]. In 8% of these patients, PTE was the direct cause of mortality [10]. In addition to its rarity, pulmonary tumor embolisms are difficult to detect and heterogenous in their presentation. In a retrospective study conducted by He et al, radiological studies did not detect the majority of PTEs and most of the patients with PTE on autopsy as a cause of death had no clinical symptoms [11]. These findings demonstrate the incredibly subtle yet dire nature of PTEs.

Two other case reports were found describing PTE secondary to RCC. Manley et al. [12] described a case of a clinically deteriorating man where evidence of PTE has only discovered post-mortem. Ogawa et al. described a case of an acute presentation of dyspnea where a PTE was confirmed using FDG-PET/CT [13]. In the latter, the patient already had a significant tumor thrombus extending from the renal primary tumor into the infradiaphragmatic inferior vena cava prior to developing a PTE [13]. However in our case, dissimilar to the others, the PTE developed with no active RCC and years into remission following a right nephrectomy and pazopanib therapy. For this reason, we postulate that the patient's lingual embolism arose from either tumor thrombotic microangiopathy or a microscopic tumor embolism. Undetectable by imaging, we speculate it grew insidiously until it became large enough to cause the patient's dyspnea.

To our knowledge, this is the first case report of a PTE secondary to RCC detected by PSMA-PET/CT. It underscores the importance of high-quality novel PSMA radiotracers and the clinical utility of CECT in hybrid imaging for detecting incidental findings. But most importantly, it alerts even the most senior clinicians to judiciously assess CECT and consider the possibility of PTEs in the context of pulmonary PSMA-avidity for RCC patients, especially if they are dyspneic.

## Conclusion

The development of novel PSMA-targeting agents in PET/CT has empowered imaging specialists and oncologists with profound diagnostic capability, but in doing so, it opens the doors to a multitude of previously unconsidered diagnoses. As demonstrated in this case, the finding of PSMA-avidity in the lungs is not limited to only parenchymal metastases. It highlights that if there is pulmonary PSMA-avidity on PET, especially in a patient with known RCC and dyspnea, it should prompt the clinician to evaluate the CECT to assess for nonmetastatic findings, including but not limited to PTE.

## Patient consent

Written, informed consent was obtained from the patient under the knowledge that their case would be described in this publication.
